# Tongxinluo Enhances Neurogenesis and Angiogenesis in Peri-Infarct Area and Subventricular Zone and Promotes Functional Recovery after Focal Cerebral Ischemic Infarction in Hypertensive Rats

**DOI:** 10.1155/2016/8549590

**Published:** 2016-03-16

**Authors:** Li Chen, Xiaoting Wang, Jian Zhang, Chao Dang, Gang Liu, Zhijian Liang, Gelun Huang, Weijia Zhao, Jinsheng Zeng

**Affiliations:** ^1^Department of Neurology, The First Affiliated Hospital, Guangxi Medical University, Nanning, Guangxi Zhuang Autonomous Region 530021, China; ^2^Guangxi Key Laboratory of Regenerative Medicine and Guangxi Collaborative Innovation Center for Biomedicine, Guangxi Medical University, Nanning, Guangxi Zhuang Autonomous Region 530021, China; ^3^Department of Neurology, Wuzhou Red Cross Hospital, Wuzhou, Guangxi Zhuang Autonomous Region 543002, China; ^4^Department of Neurology and Stroke Center, The First Affiliated Hospital, Sun Yat-Sen University, Guangzhou, Guangdong 510080, China

## Abstract

*Background*. Tongxinluo is a traditional Chinese medicine compound with the potential to promote the neuronal functional recovery in cerebral ischemic infarction.* Objective*. This study aimed to disclose whether tongxinluo promotes neurological functional recovery and neurogenesis and angiogenesis in the infarcted area and SVZ after cerebral ischemic infarction in hypertensive rats.* Methods.* The ischemic model was prepared by distal middle cerebral artery occlusion (MCAO) in hypertensive rats. Tongxinluo was administrated 24 h after MCAO and lasted for 3, 7, or 14 days. Behavioral tests were performed to evaluate the protection of tongxinluo. Immunochemical staining was applied on brain tissue to evaluate the effects of tongxinluo on neurogenesis and vascularization in the MCAO model rats.* Results*. Postinjury administration of tongxinluo ameliorated the neuronal function deficit in the MCAO model rats. As evidenced by the immunochemical staining, BrdU^+^/DCX^+^, BrdU^+^/nestin^+^, and BrdU^+^ vascular endothelial cells were promoted to proliferate in SVZ after tongxinluo administration. The matured neurons stained by NeuN and vascularization by laminin staining were observed after tongxinluo administration in the peri-infarct area.* Conclusion.* Tongxinluo postischemia administration could ameliorate the neurological function deficit in the model rats. Possible mechanisms are related to neurogenesis and angiogenesis in the peri-infarct area and SVZ.

## 1. Introduction

The mechanisms underlying cerebral injury have been advanced. Cerebral infarction leads to neuropathological deficits at the ischemic site possibly through eliciting excitoxicity, neuroinflammation, and apoptosis of functional neurons [1–3]. Interestingly, neurogenesis is an effective approach to facilitate the neurological recovery after cerebral ischemic injury [4–6]. Tongxinluo is characterized by multiefficacies because of its abundant active components. As reported, tongxinluo possesses activities of antioxidation and vulnerable plaque stabilization [7]. Therefore, tongxinluo likely ameliorates the symptoms after cerebral infarction by different mechanisms. We previously reported that administration of tongxinluo 24 h after cortical infarction promoted neurogenesis and angiogenesis in the ipsilateral thalamus [8]. However, ipsilateral thalamus is not the direct infarcted area and the effect of tongxinluo on this second injury after ischemia injury might not be completely similar to that in the infarcted area. In this study, distal middle cerebral artery occlusion (MCAO) in renovascular hypertensive rats was modeled to investigate whether tongxinluo facilitates neurogenesis and angiogenesis in the infarcted area and subventricular zone (SVZ).

## 2. Materials and Methods

### 2.1. Materials and Reagents

The tongxinluo powder was obtained from Yiling Pharmaceutical Incorporated Company (Shijiazhuang, China). Generally, the prescription comprises 12 components. All the components were authenticated and standardized according to the markers described in the Chinese Pharmacopoeia 2005 (National Pharmacopoeia Committee, 2005). In addition, the ingredients were strictly standardized as previously described [9, 10].

### 2.2. Animal Model and Treatment

All the experimental procedures were approved by the Animal Research Ethics Committee of the First Affiliated Hospital of Sun Yat-Sen University. Efforts were made to minimize the number of animals used.

Sixty male Sprague-Dawley rats weighing 70 to 90 g were utilized to produce stroke-prone renovascular hypertensive rats (RHRSP) according to the method previously described [11]. Briefly, the rats were anesthetized with 10% chloral hydrate (3 mL/kg, intraperitoneal injection, i.p.) and underwent a surgery to induce bilateral renal artery constriction by silver clips. Systolic blood pressure was under monitoring once weekly with a tail-cuff sphygmomanometer (ML866 PowerLab 4/30; AD Instruments Pty Ltd., Sydney, Australia). Twelve weeks after the operation, 54 rats with systolic blood pressure of > 180 mmHg were assigned to receive permanent distal MCAO.

The MCAO model in the hypertensive rats was produced as previously described [12]. Briefly, rats were anesthetized with 10% chloral hydrate through an i.p. injection. With the help of an operating microscope, the right middle cerebral artery (MCA) of rats was exposed and then occluded above the olfactory tract and distal to the striatal branches by microbipolar coagulation. The 48 rats with successful MCAO were randomly divided into two groups: vehicle and tongxinluo group (*n* = 24 per each group). The other six RHRSP rats were excluded from this study due to failed MCAO, intracranial hemorrhage, or death during the experiment. Rats were sacrificed 3, 7, or 14 days after MCAO (*n* = 8 at each time point).

In the tongxinluo group, the rats were intragastrically administrated once daily at a volume of 3 mL/kg (0.5 g/kg/day). The administration was started at 24 h after MCAO for 3, 7, or 14 days. As control, the rats were treated with an equal volume of water through similar administration. The dosage of tongxinluo was chosen based on our previous study [8]. For the proliferation test, BrdU (50 mg/kg, Sigma-Aldrich, USA) was injected intraperitoneally twice daily for 3 or 6 consecutive days initiating from 24 h after MCAO in all rats.

### 2.3. Neurological Functional Assessment

Behavioral testing and scoring were assessed blindly by an experimenter. Neurological scores were performed 3, 7, or 14 days after MCAO right before decapitation. Bederson scores were used to evaluate global neurological function as previously described [8]. Briefly, the rats were suspended by the tail at 20 cm above the floor. The animals were scored based on the symptoms of the rats: (1) a normal response with extension of both forelimbs toward the floor was scored as 0; (2) resistance to sliding in both directions when lateral pressure was applied from behind the shoulders was scored as 1; (3) reduced resistance to a lateral push from the paretic side was scored as 2; (4) spontaneous circling toward the paretic side or left-sided tumbling or unmoving was scored as 3.

### 2.4. Infarction Volume Measurement

Nissl staining was performed to measure infarct volume 3, 7, or 14 days after MCAO. The relative infarct volume is calculated as the percentage of the contralateral hemisphere.

### 2.5. Immunohistochemistry

A series of coronal sections were selected to perform immunochemical staining of BrdU, nestin, DCX, NeuN, and laminin. The primary antibodies were applied including sheep polyclonal anti-BrdU (1 : 500, Abcam, USA), mouse monoclonal anti-BrdU (1 : 1000, Sigma-Aldrich, USA), mouse monoclonal anti-nestin (1 : 1000, Chemicon, USA), mouse monoclonal anti-neuronal nuclei (NeuN, 1 : 1000, Chemicon, USA), or rabbit polyclonal anti-laminin (1 : 200, Sigma-Aldrich, USA). After blocking with goat serum and incubation with primary antibodies overnight at 4°C, the slices were incubated with fluorescent-labeled secondary antibodies Alexa Fluor 488-conjugated anti-sheep IgG (1 : 200), Cy3-conjugated anti-mouse IgG (1 : 200), or Cy3-conjugated anti-rabbit IgG (1 : 200; all three antibodies from Jackson Immunoresearch Laboratories, USA) for 1 h at room temperature. For BrdU immunofluorescence, brain sections were pretreated for 30 min at 37°C with 2 N HCl and then rinsed for 10 min in 0.1 M boric acid (pH = 8.5) at room temperature followed by incubation with blocking solution. Phosphate-buffered saline instead of primary antibody was applied in negative control. The images were taken from four different fields at least (Olympus BX51; Olympus).

## 3. Quantification for the Staining

The immunochemical staining images were taken and quantified by an experimenter who had no knowledge of the groups. The staining was analyzed by Image-Pro Plus image analysis software (Media Cybernetics, Silver Spring, USA). A parameter of bregma between −3.0 and −1.2 mm was set in the data analysis. Every sixth section from a series of coronal sections was selected. The numbers of double-positive cells were counted within three nonoverlapping fields (425 × 320 *μ*m^2^) at 400x magnification. The average data from each field from each section are presented. For measurement of vascular density in the peri-infarction cortex, images were digitized within the field (850 × 640 *μ*m^2^) at 400x magnification and data was expressed as the percentage of area expressing laminin.

### 3.1. Statistical Analyses

The data are presented as the mean ± standard error of mean (SEM). One-way ANOVA was used for three-group comparison. Values from neurological functional performance are expressed as the median and quartile range and were analyzed with Kruskal-Wallis tests. Statistical analysis was performed with SPSS 13.0 for Windows (SPSS Inc., Chicago, IL, USA). *p* < 0.05 was set as statistically significant.

## 4. Results 

### 4.1. Tongxinluo Improves Neurological Function

The neurological function was tested by Bederson score. All MCAO rats exhibited progressive recovery during the experimental days. The Bederson score was gradually decreased in the vehicle group over time. A similar trend was observed in the tongxinluo treatment group. However, compared with corresponding time point in vehicle group (day 7 and day 14), tongxinluo treatment significantly improved the behavioral outcome (Table 1, *p* < 0.05). The infarct volume was also detected in the two groups. The decrease of infarct volume was apparent in both vehicle and tongxinluo groups over time, which was consistent with the behavioral alterations. However, there was no significant difference of infarct area between the tongxinluo and vehicle groups at the observed time points (Table 1, *p* < 0.05).

### 4.2. Tongxinluo Improves Proliferation and Migration of Endogenous Neural Stem Cells in the SVZ Region

**Figure 1:**
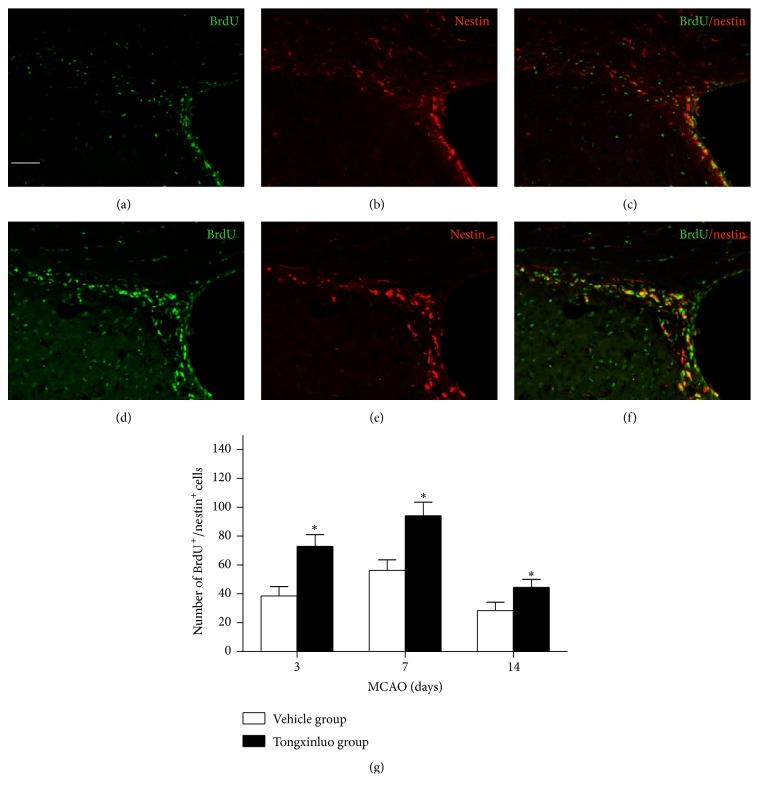
Tongxinluo enhances proliferation and migration of endogenous neural stem cells in the SVZ. (a), (b), and (c) are the representative images of BrdU, nestin, and merge in the dorsolateral ventricle wall in vehicle group. (d), (e), and (f) are the representative images of BrdU, nestin, and merge in the dorsolateral ventricle wall in tongxinluo group. (g) Quantification data of BrdU^+^/nestin^+^ cells in SVZ. ^*∗*^ *p* < 0.05 corresponding to vehicle group. Scale bar: 100 *μ*m.

Nestin is an intermediate filament protein which is typically expressed in neural stem/progenitor cells. To examine the effect of tongxinluo treatment on proliferation of neural stem cells, we identified newly born neural stem/progenitor cells by BrdU and nestin double labeling. Compared with vehicle, tongxinluo treatment significantly increased the percentage of BrdU^+^/nestin^+^ cells in the SVZ region (Figure 1, *p* < 0.05). The peak was found at day 7 after tongxinluo treatment. These results suggest that tongxinluo enhances neural stem cell proliferation after ischemic injury.

**Figure 2:**
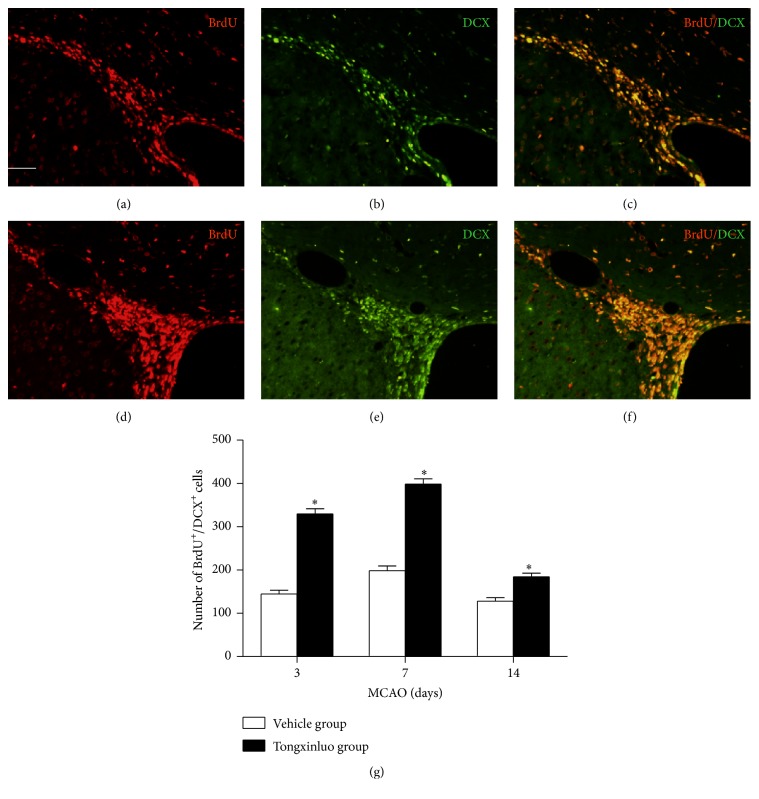
Tongxinluo enhances proliferation and migration of endogenous neural stem cells in the SVZ. (a), (b), and (c) are the representative images of BrdU, DCX, and merge in the dorsolateral ventricle wall in vehicle group. (d), (e), and (f) are the representative images of BrdU, DCX, and merge in the dorsolateral ventricle wall in tongxinluo group. (g) Quantification data of BrdU^+^/DCX^+^ cells in SVZ. ^*∗*^ *p* < 0.05 corresponding to vehicle group. Scale bar: 100 *μ*m.

Doublecortin (DCX) is a microtubule-associated protein which is widely expressed on migrating neuroblasts. To examine the effect of tongxinluo treatment on neuroblasts, we measured the percentage of BrdU and DCX double-positive cells. Compared with vehicle treatment, tongxinluo treatment significantly increased the percentage of BrdU^+^/DCX^+^ cells in the SVZ (Figure 2, *p* < 0.05). These results further suggest that tongxinluo promotes neurogenesis after ischemic injury.

### 4.3. Tongxinluo Enhances Differentiation of Endogenous Neural Stem Cells in the Peri-Infarct Area

**Figure 3:**
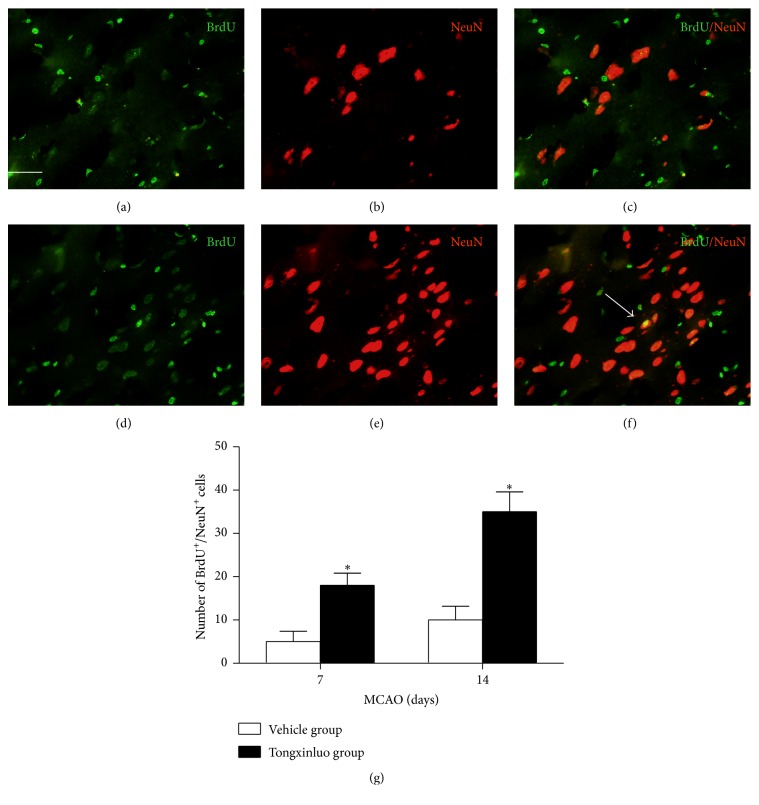
Tongxinluo enhances NeuN positive cells in the infarcted area. (a), (b), and (c) are the representative images of BrdU, NeuN, and merge in the infarcted area in vehicle group. (d), (e), and (f) are the representative images of BrdU, NeuN, and merge in the infarcted area in tongxinluo group. (g) Quantification data of BrdU^+^/NeuN^+^ cells in the infarcted area. ^*∗*^ *p* < 0.05 corresponding to vehicle group. Scale bar: 50 *μ*m.

To further investigate whether proliferative neural stem/precursor cells differentiate into mature neuronal cells within the infarcted area, we performed double immunofluorescent staining of BrdU and NeuN. As shown in Figure 3, treatment with tongxinluo apparently increased BrdU^+^/NeuN^+^ cells in the peri-infarct area 14 days after MCAO.

### 4.4. Tongxinluo Increases Angiogenesis in the SVZ and Peri-Infarct Area

**Figure 4:**
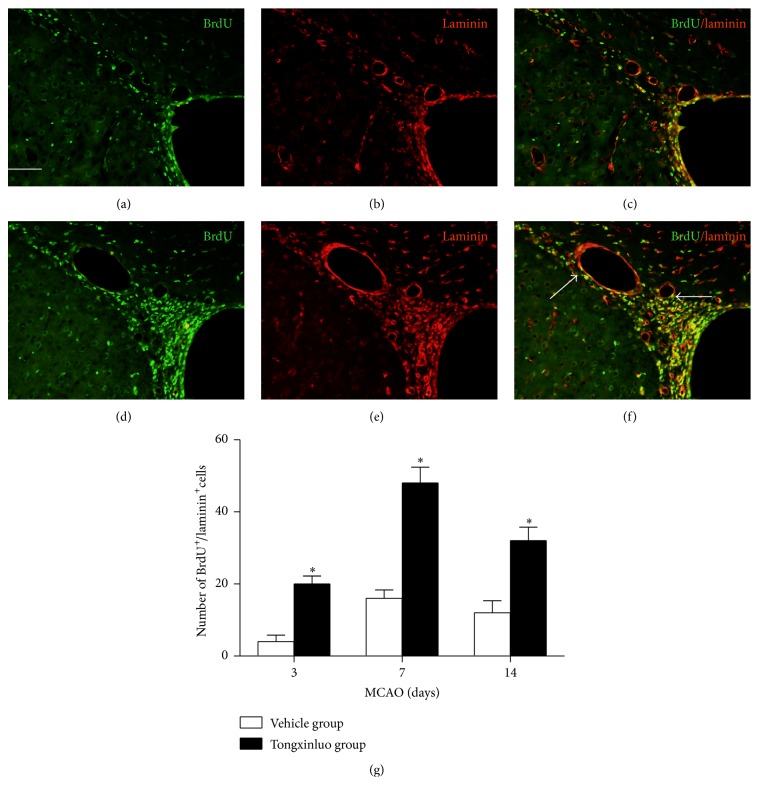
Angiogenesis is associated with endogenous neurogenesis in the SVZ in tongxinluo group. (a), (b), and (c) are the representative images of BrdU, laminin, and merge of SVZ in the dorsolateral ventricle wall in vehicle group. (d), (e), and (f) are the representative images of BrdU, laminin, and merge in the dorsolateral ventricle wall in tongxinluo group. (g) Quantification data of BrdU^+^/laminin^+^ cells in SVZ. ^*∗*^ *p* < 0.05 corresponding to vehicle group. Scale bar: 100 *μ*m.

**Figure 5:**
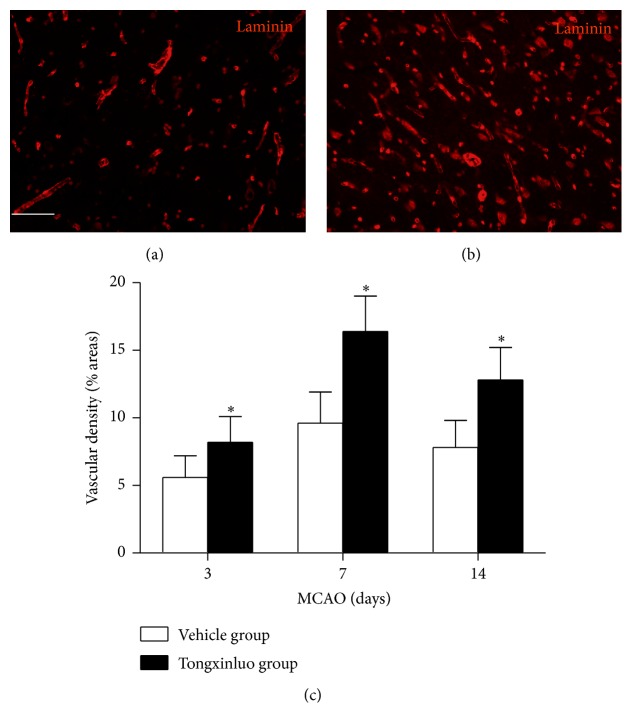
Tongxinluo enhances vascular density in the infarcted area. (a) and (b) are the representative images of laminin staining in vehicle group and tongxinluo group. (c) Quantification data of laminin cells in the infarcted area. ^*∗*^ *p* < 0.05 corresponding to vehicle group. Scale bar: 100 *μ*m.

As the microenvironment plays critical roles to support the neurogenesis, we detected the laminin expression in the SVZ region and peri-infarct area. As shown in Figure 4, treatment with tongxinluo apparently increased newly born vascular endothelial cells which were labeled with BrdU^+^/laminin^+^ double staining in SVZ region. In addition, rats that received tongxinluo exhibited a significant increase in vascular density in peri-infarct area (Figure 5, *p* < 0.05). The peak time window was consistent with that observed in neurogenesis experiment. These data suggest that tongxinluo promotes angiogenesis after ischemic injury.

## 5. Discussion

As is known, the undisclosed mechanisms restrict the internationalization of TCMs. Even some excellent medicines with clear clinical effects in specific diseases were still not accepted for the disease treatment. Therefore, a clear detail describing the mechanisms underlying the therapeutic effect of TCM is urgently necessary. In this study, we showed that postinjury administration of tongxinluo ameliorated the neuronal function deficit in the ischemic injury model. The possible mechanisms were related to the neurogenesis in the subventricular zone (SVZ) region. Both DCX^+^ and nestin^+^ cells were promoted to proliferate after tongxinluo administration and peaked at day 7. The matured neurons stained by NeuN and vascularization by laminin staining were observed after tongxinluo administration in the peri-infarct area.

Tongxinluo comprises 12 components:* Borneolum Syntheticum*,* Panax ginseng*,* Ziziphus jujuba and Z. spinosa, Paeonia lactiflora, Santalum album, Dalbergia odorifera, Boswellia carteri *Birdw.,* Scolopendra subspinipes mutilans, Buthus martensii *Karsch,* Hirudo nipponica *Whitman,* Cryptotympana pustulata *Fabricius, and* Steleophage plancyi* [13]. The protective effect of tongxinluo on myocardial ischemia-reperfusion injury was well studied. A variety of signaling pathways including PKA and ERK pathways were supposed to regulate the protective effect of tongxinluo in myocardial ischemic injury [14–16]. In this study, a MCAO model using RHRSP rats was established to disclose the protective effect of tongxinluo on cerebral ischemic injury. As patients with ischemic stroke always experience the symptom of hypertension, the model in our study has a close similarity to the clinical practice. Importantly, the model has high reproducibility because the lesion is restricted locally in the cortex. Although we previously reported the protection of tongxinluo against the secondary injury elicited by ischemic injury in the primary locus, in that study, the protective mechanism in the infarcted area was not disclosed. Here, the neuronal function was evaluated by Bederson test. A clear decrease of the scores in 3, 7, and 14 days after treatment indicates the self-recovery after ischemic injury. Correspondingly, the infarcted area was also reduced over time. By contrast, the Bederson score was much lower in tongxinluo treated group compared with vehicle at day 7 and day 14 after injury, which implicates that tongxinluo promotes the recovery following focal cerebral ischemic injury. However, the infarcted area after ischemia injury was not altered by tongxinluo application. This result was due to the fact that postinjury application of tongxinluo did not affect the formed infarcted area. In combination with our previous report [8], this study further supports the neuroprotection of tongxinluo. In fact, the protective role of tongxinluo on cerebral ischemic injury was also reported by the other lab. However, in those studies, schemes with preapplication or pre-post administration of tongxinluo were applied, which was different from our postapplication of tongxinluo [10, 17]. Although the infarcted area was decreased with tongxinluo preapplication, the preapplication of drug only showed preventive effect in the disease, but not therapeutic effect. Our study suggested that tongxinluo also has a therapeutic effect against the symptoms caused by ischemic injury.

Ischemic injury will signal the stem cell populations in the adult brain to divide, and to send immature neurons to damaged areas [18]. However, the numbers of new cells are insufficient to repair the injury elicited by ischemic injury [19]. Scientists tried to find new approaches to facilitate the increase of matured functional neurons in the infarcted area [20, 21]. A number of positive results were reported regarding the protective effects of chemical compounds [20, 22]. Furthermore, the TCMs were prominent in facilitating the neurogenesis [8, 23]. Transplantation of exogenous cultured stem cell into the infarcted area was proposed to increase the numbers of functional neurons and promote the functional recovery [24]. However, the survival of the transplanted cells in the infarcted area is still a big challenge due to the rejection reaction [25]. In this study, we demonstrated that tongxinluo was able to increase the proliferation of endogenous neural stem cells in the SVZ region. The BrdU^+^/DCX^+^ double-positive cells were significantly increased at day 3 after tongxinluo treatment. This increase was even apparent at day 7 after tongxinluo application and then gradually decreased at day 14. These results implicate that 14 days after injury is the optimal time window to improve the neuronal stem cell proliferation. These results were further confirmed by the costaining of BrdU and nestin.

The increase of BrdU^+^ cells as well as BrdU^+^/DCX^+^ or BrdU^+^/nestin^+^ cells only implicates a potential of neurogenesis after tongxinluo in the infarcted area. The functional neurons after ischemia injury will ameliorate the functional recovery in the model. For this reason, we distinguished the BrdU^+^/NeuN^+^ cells in the infarcted area. Tongxinluo could also increase these populations of cells. These data further support the notion that endogenous neurogenesis is an important and efficient approach to promote the functional recovery after ischemic injury.

The microenvironment is critical for the stem cell self-renewal and specific differentiation [26]. Blood and oxygen deficiency and release of inflammation reactive factors and apoptosis inducer inhibit the survival of the new proliferated stem cells. A harmony microenvironment promotes the endogenous stem cell renewal and decreases the apoptosis of the stem cells [26]. In fact, the metabolism of the microenvironment is supported by the new vascular system established. In the ischemic injury model, angiogenesis was increased after tongxinluo application. The laminin expression was peaked at day 7 after injury. The dynamic alteration of laminin was corresponding to neurogenesis after tongxinluo administration. Hence, the angiogenesis after tongxinluo administration likely helps to clean the metabolites or the residual toxic substances in the infarcted area. Importantly, the nutritional supplement will promote stem cell specific differentiation or facilitate the establishment of new synaptic connections.

Under physiological and pathological conditions, TCM has the typical advantages in the disease treatment, including overall adjustment, bidirectional regulation, and multiple prevention-treatment-repairing properties. In other words, TCM has multitarget actions, rather than single effect. The overall view in TCM is completely different from the western medical system. As we know, multiple components in tongxinluo prescription have the capacity to increase the neurogenesis after ischemic injury, such as ginseng total saponins in Radix Ginseng [27]. Additionally, the active compounds which can promote the angiogenesis are also included in the prescription. However, the function exhibition of tongxinluo should be attributable to the overall prescription. Regarding the protective effects of tongxinluo, we think that all these roles are related to blood circulation and stasis resolving effect. Tongxinluo functions through circulating the cerebral blood and eliminating the metabolic waste products in the brain to maintain balance and harmony of the internal environment, which was critical for the neural regeneration.

## 6. Conclusion

In this study, a distal MCAO in hypertensive rat model was produced to verify the symptom relieving effects of tongxinluo. The postinjury administration of tongxinluo could ameliorate the neuronal functional deficit in MCAO model rats. Possible mechanisms are related to neurogenesis and angiogenesis in the SVZ and peri-infarct area. This study suggests that tongxinluo is a candidate drug for therapeutics of ischemic injury.

## Figures and Tables

**Table 1:** Tongxinluo treatment ameliorated neurological function without affecting infarct volume.

	Day 3	Day 7	Day 14
Vehicle			
Bederson score	2.75 (2.5, 3)	2.5 (2.25, 2.75)	1.5 (1.25, 1.75)
Infarct volume	24.85 ± 2.78%	19.36 ± 2.96%	16.64 ± 1.62%
Tongxinluo			
Bederson score	2.75 (2.25, 3)	1.5 (1.25, 2)^*∗*^	1.0 (1, 1.75)^*∗*^
Infarct volume	25.56 ± 2.44%	20.62 ± 2.25%	17.58 ± 1.58%

MCAO: middle cerebral artery occlusion.

Values are mean ± SEM or median and quartile range from six rats. ^*∗*^ *p* < 0.05 compared with the vehicle group.
